# Dataset of assembly and annotation of the mitogenomes of *Triatoma dimidiata* and *Triatoma huehuetenanguensis* captured from Yucatán, México

**DOI:** 10.1016/j.dib.2023.109866

**Published:** 2023-12-07

**Authors:** Nohemi Cigarroa-Toledo, Carlos M. Baak-Baak, José I. Chan-Pérez, David I. Hernandez-Mena, Karla C. Amaya Guardia, Maria F. Ocaña-Correa, Angelica Pech-May, Karla Y. Acosta-Viana

**Affiliations:** a Laboratorio de Biología Celular, Centro de Investigaciones Regionales ‘‘Dr. Hideyo Noguchi’’, Universidad Autónoma de Yucatán, Avenida Itzaes, No. 490 x Calle 59, Col. Centro, Mérida, Yucatán, C.P. 97000, México; b Laboratorio de Arbovirología, Centro de Investigaciones Regionales ‘‘Dr. Hideyo Noguchi’’, Universidad Autónoma de Yucatán, Calle 43 s/n x 96, Col. Paseo de las Fuentes, Mérida, Yucatán, C.P. 97225, México; c Centro de Investigación y de Estudios Avanzados, Instituto Politécnico Nacional, Unidad Mérida, Antigua Carretera Progreso Km 6, Cordemex, Mérida, Yucatán, 97310, México; d Laboratorio de Chagas, Centro Regional de Investigaciones en Salud Pública, Tapachula, Chiapas, México

**Keywords:** Mitochondrial genome, Kissing bug, Chagas disease

## Abstract

*Triatoma dimidiata* is a species complex, and its members are responsible for the transmission of *Trypanosoma cruzi*, the causative agent of Chagas disease. We present the assembly and annotation of the mitogenome of the *Triatoma dimidiata* (Latreille, 1811) and *Triatoma huehuetenanguensis* Lima-Cordón & Justi, 2019. The mitochondrial genomes were successfully sequenced using the Illumina Nextseq 500 platform, 2×75 cycles, and 5 million reads per sample. Contigs were assembled and annotated using the reference genomes of *T. dimidiata* and *T. huehuetenanguensis* available in Genbank (NC_002609 and NC_050325.1, respectively). The mitogenomes of *T. dimidiata* have lengths of 17,008 bp, while those of *T. huehuetenanguensis* are 15,910 bp and 15,909 bp. The genome comprises 13 protein-coding genes, 22 transfer RNA genes, two ribosomal RNA genes, and a control region. The mitogenomes will be valuable to scholars and students focused on integrative taxonomy, phylogeography, and evolutionary studies of the *Triatoma dimidiata* complex and the transmission of Chagas diseases.

Specifications Table

**Table:** 

Subject	Omics: genomics.
Specific subject area	*Triatoma dimidiata* complex, Mitogenomics
Data format	Raw sequencing reads and the assembled and annotated mitogenomes.
Type of data	FASTQ: DNA sequence reads. FASTA: mitochondrial genome assembly. FIGURE: mitogenomic circular map.
Description of data collection	Genomic DNA was extracted from legs and head of Triatomines using the DNeasy^Ⓡ^ Blood & Tissue Kit (QIAGEN, Hilden, Germany). Three whole mitogenome shotgun libraries were sequenced on an Illumina Nextseq 500 platform using the paired-end protocol (2×75 cycles, 5 million reads per sample). The mitogenomes of *T. dimidiata* and *T. huehuetenanguensis* were assembled with Geneious Prime software using reference genomes available in Genbank. Two tools were used to annotate the genomes: MITOS WebServer and Geneious Prime software. OGDRAW was used to generate the circular mitochondrial genome map.
Data source location	The triatomines were captured in houses. *T. dimidiata* was captured in Choyob, and *T. huehuetenanguensis* in Hubila, Tixkokob, and Mérida Yucatán, México. The specimens were transported alive to the Cell Biology Laboratory at the Universidad Autónoma de Yucatán, México, where the species were observed by stereoscope and the genomic DNA was extracted.
Data accessibility	Repository name: Triatoma dimidiata Genome sequencing and assembly Data identification number: PRJNA1018562 Direct URL to data: http://www.ncbi.nlm.nih.gov/bioproject/1018562 Repository name: NCBI Genbank Data identification number: OR611930, OR611931, OR611932

## 1 Value of the Data

• The sequences represent the first mitogenomes assembled and annotated by *T. dimidiata* and *T. huehuetenanguensis* in Yucatán, in southeastern México.
• The mitogenomes can benefit researchers and students working on triatomine systematics, phylogeography, and evolution.
• The mitogenomes are valuable for training phylogenetic models of triatomines, especially for better understanding the speciation of the *Triatoma dimidiata* complex.

## 2 Data Description

*Triatoma dimidiata* (Latreille, 1811) (Hemiptera, Reduviidae) is a species complex, and its members are vectors of *Trypanosoma cruzi*, the causal agent of Chagas disease [1]. Implementing control measures requires accurate identification of the triatomine involved in the transmission cycles. Phylogenetic studies have defined three haplogroups of *T. dimidiata* in México [2]. Before this study, the presence of *Triatoma nitida* and two haplogroups of *Triatoma dimidiata* (H1 and H2) were known in Yucatán. The presence of *T. huehuetenanguensis* in the state was hypothesized, we verified its occurrence in the southeast of México [3].

## 3 Experimental Design, Materials and Methods

### 3.1 Collection of triatomine adults

In April 2022, adult triatomines were captured in houses in Yucatán, México. In the rural community of Choyob, Muna; in Hubila, Tixkokob; and in the urban city of Mérida, Yucatán, México. The specimens were transported alive to the Cell Biology Laboratory at the Universidad Autónoma de Yucatán (UADY), México and were identified under a stereoscope (Leica Microsystems, Switzerland), with the help of dichotomous keys. The species was identified following the morphological characteristics described by Lent and Wygodzinsky [4]. The project was reviewed and approved by the Research Advisory Committee and the Research Ethics Committee of the Centro de Investigaciones Regionales “Dr. Hideyo Noguchi”, UADY, and was registered with the number CIRB-2021-0008.

### 3.2 DNA sampling and sequencing

DNA was isolated from three adult female. Each specimen´s legs and head were placed in a mortar and crushed with liquid nitrogen. The macerated tissue was used to extract the genomic DNA, following the manufacturer´s protocol for the DNeasy^Ⓡ^ Blood & Tissue Kit (QIAGEN, Hilden, Germany). DNA was quantified in a Nanodrop 2000 (Thermo Scientific^Ⓡ^), and integrity was visualized in 1.0% agarose gel stained with ethidium bromide and visualized on a Doc™ XR+ Gel Documentation System by Bio-Rad Laboratories, Inc.

Subsequently, 30 µl DNA (6 ng/µl) of each sample were used for sequencing. The construction and sequencing of mtDNA libraries were performed on the Illumina Nextseq 500 platform (2×75 cycles, 5 million reads/sample) at the Unit of Massive Sequence and Bioinformatics of the Instituto de Biotecnología, Universidad Nacional Autónoma de México, México. The sequencing runs produced an average of 1,733,096, 1,652,733, and 1,598,505 million (M) reads in *T. dimidiata* (*n* = 1) and *T. huehuetenanguensis* (*n* = 2), respectively, with a quality score greater than 34 (Q34), corresponding to a 99.9% base call accuracy.

### 3.3 Mitogenome assembly and feature analysis

The FASTQ read files (sequences) were imported and matched into Geneious Prime v. 2022.2.2, where the reads were subsequently filtered and trimmed using the plugin BBDuk (Decontamination Using Kmers), which is a fast and accurate tool for trimming and filtering NGS reads. This plugin allowed the trimming of low-quality Illumina adapters and ends and discarte short reads that were below a minimum length. To assemble the mitochondria of our specimens, the reads were mapped with the Geneious algorithm (sensitivity: Medium Sensitivity/Fast), using the mitochondria of *T. dimidiata* (NC_002609) published in Genbank as reference sequence.

**Fig. 1:**
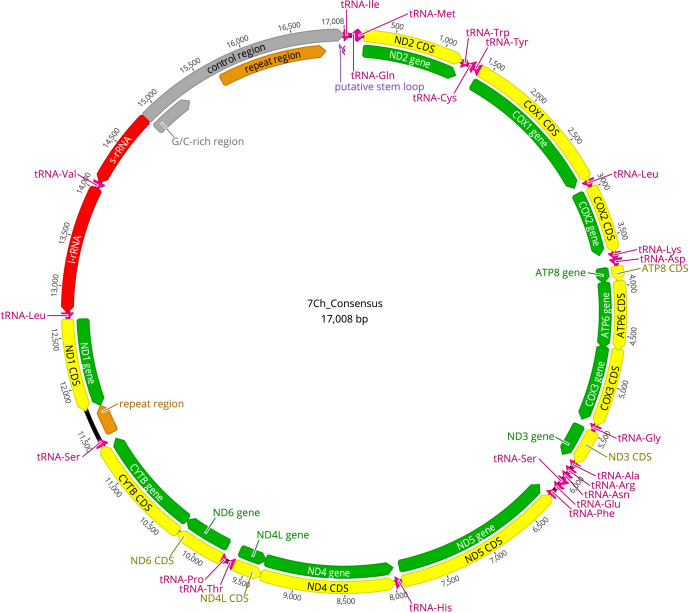
Map of the mitochondrial genome of *Triatoma dimidiata* (17,008 bp). The annotations of the protein genes in green and the CDS in yellow, the ribosomal RNA coding regions in red, the transfer RNA in pink, and the repeating regions in orange are indicated. The abbreviations for the genes are COX1, COX2, and COX3, which refer to the cytochrome oxidase subunits; CYTB refers to cytochrome B; and ND1 to ND6 refer to NADH dehydrogenase components; tRNAs are labeled according to the “three-letter code”. The orientation of the arrows indicates the direction of transcription for each of the respective coding regions. (For interpretation of the references to color in this figure legend, the reader is referred to the web version of this article.)

**Fig. 2:**
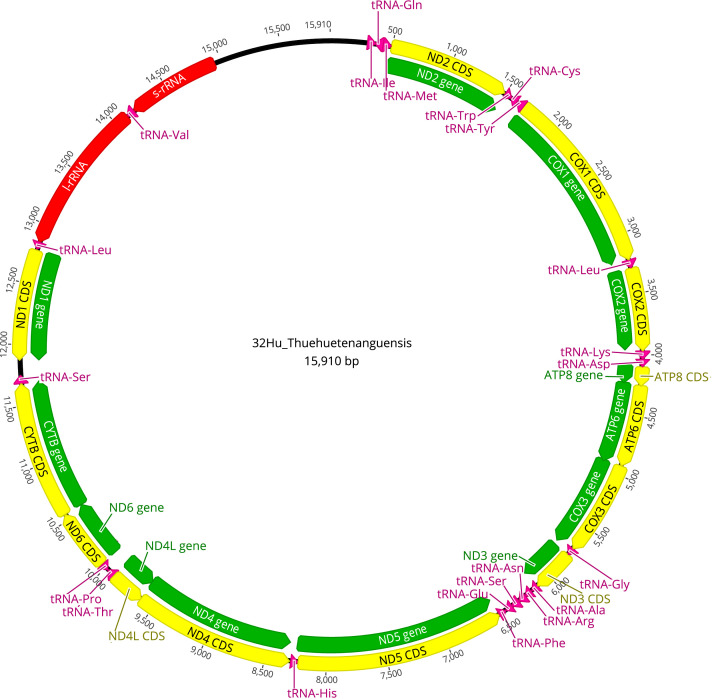
Map of the mitochondrial genome of *Triatoma huehuetenanguensis* (15,910 bp). The annotations of the protein genes in green and the CDS in yellow, the ribosomal RNA coding regions in red, and of the transfer RNA in pink are indicated. The abbreviations for the genes are COX1, COX2, and COX3, which refer to the cytochrome oxidase subunits; CYTB refers to cytochrome B; and ND1 to ND6 refer to NADH dehydrogenase components; tRNAs are labeled according to the “three-letter code”. The orientation of the arrows indicates the direction of transcription for each of the respective coding regions. (For interpretation of the references to color in this figure legend, the reader is referred to the web version of this article.)

**Fig. 3:**
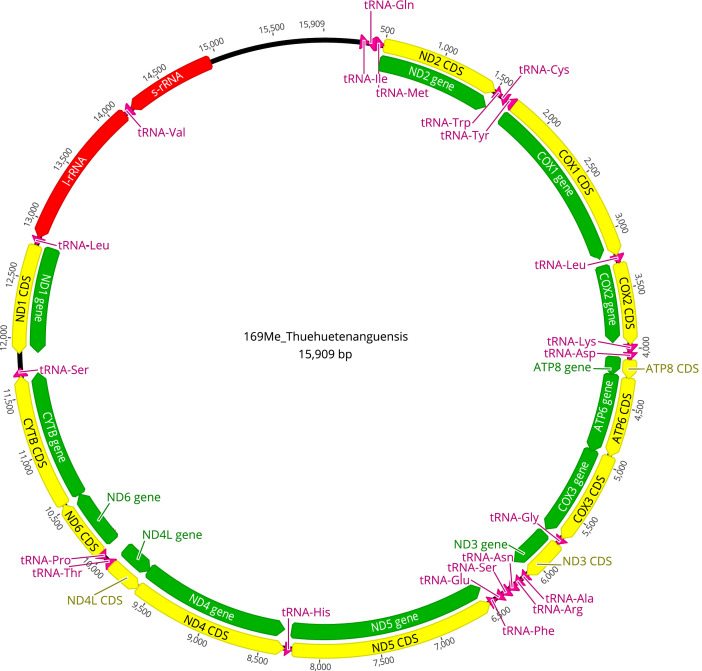
Map of the mitochondrial genome of *Triatoma huehuetenanguensis* (15,909 bp). The annotations of the protein genes in green and the CDS in yellow, the ribosomal RNA coding regions in red, and of the transfer RNA in pink are indicated. The abbreviations for the genes are COX1, COX2, and COX3, which refer to the cytochrome oxidase subunits; CYTB refers to cytochrome B; and ND1 to ND6 refer to NADH dehydrogenase components; tRNAs are labeled according to the “three-letter code”. The orientation of the arrows indicates the direction of transcription for each of the respective coding regions. (For interpretation of the references to color in this figure legend, the reader is referred to the web version of this article.)

Once we obtained the complete sequence of the mitochondria, we annotate the genes. On the one hand, an annotation was made using the Mitos2 algorithm on its web server (http://mitos2.bioinf.uni-leipzig.de/index.py), using the parameters “Reference: Refseq 63 Metazoa” and “Genetic Code: 5 Invertebrate” and with Geneious Prime software the *T. dimidiata* (NC_002609) genome was used as a reference to annotate genes. The consensus sequences obtained in this study was aligned with the reference genome (NC 002609) using the “Geneious Alignment” tool. Once the mitochondrial genomes were aligned, the NC_002609 annotations were transferred to our genome, with a similarity percentage of at least 80%. A comparison was made between the annotations obtained in Mitos2 and in Geneious Prime, and since they completely coincided, we chose to present the one from Mitos2. Furthermore, the MITOS mitochondrial genome annotations were manually curated by aligning the predicted genes via the web service of NCBI BLAST [5] (with default parameters, version of February 2023) to gene annotations for *T. dimidiata* (NC_002609) and *T. huehuetenanguensis* (NC_050325.1). The sequences of each mitochondrial genome obtained in this study were identified by BLASTn alignments [6] “Organism Optional” Triatominae (taxid:70999). The mitogenomes of *T. dimidiata* have lengths of 17,008 bp, while those of *T. huehuetenanguensis* are 15,910 bp and 15,909 bp. The genome comprises 13 protein-coding genes, two rRNA genes, 22 tRNA genes, and one control region, as shown in Fig. 1, Fig. 2, Fig. 3 of the mitochondrial organization NCBI Genbank: OR611930, OR611931, OR611932.

## Limitations

Not applicable.

## Ethics Statement

This data is available in the public domain, and no funding is received for the present effort. There is no conflict of interest.

## CRediT authorship contribution statement

**Nohemi Cigarroa-Toledo:** Conceptualization, Writing – original draft, Supervision. **Carlos M. Baak-Baak:** Writing – original draft, Supervision. **José I. Chan-Pérez:** Writing – review & editing. **David I. Hernandez-Mena:** Writing – review & editing. **Karla C. Amaya Guardia:** Writing – review & editing. **Maria F. Ocaña-Correa:** Writing – review & editing. **Angelica Pech-May:** Writing – review & editing. **Karla Y. Acosta-Viana:** Writing – review & editing.

## Data Availability

TriatominGenome sequencing and assembly (Original data) (National Center for Biotechnology Information). Triatoma dimidiata Genome sequencing and assembly (Original data) (NCBI Genbank).
